# “Waking Up Every Day With the Worry”: A Mixed-Methods Study of Anxiety in Undocumented Latinx College Students

**DOI:** 10.3389/fpsyt.2020.568167

**Published:** 2020-11-13

**Authors:** Carola Suárez-Orozco, Guadalupe López Hernández

**Affiliations:** ^1^Counseling & School Psychology, University of Massachusetts Boston, Boston, MA, United States; ^2^Human Development and Psychology, University of California, Los Angeles, Los Angeles, CA, United States

**Keywords:** latinx, undocumented, college students, anxiety, social support, social belonging

## Abstract

To date, little research has taken a mixed-methods strategy to consider the ways in which living “in the shadows” without recognized legal status may affect mental health. In this study, we took this approach, to examine how legal status, as well as stressors (deportation worries, financial concerns) and potentially protective factors (faculty support, peer support), affect anxiety levels of undocumented Latinx undergraduates from colleges across California. We surveyed 486 participants including both standardized measures as well as open-ended responses. We found that rates of self-reported anxiety between undocumented females were 4 times that of the norm population and that of male undocumented students were 7 times higher as measured by the GAD-7 in the moderate and severe ranges. Our predictive models suggested that participants' rates of anxiety were in large part related to worries about financing their education and their daily living expenses as well as detainment and deportation; having an institutional agent such as a professor whom they can turn to for support served to buffer the effects of anxiety. Qualitative findings triangulated the quantitative findings and provided further insights into the experience of living with the stresses of social exclusion and liminal status.

## Introduction

Despite substantial challenges including poverty, xenophobia, and difficult work and living conditions faced by immigrants, research emerging at the end of the last century pointed to an “immigrant paradox” (1), suggesting that the first-generation typically experienced better overall physical and mental health than later immigrant generations (2–4). Subsequent research began to unpack nuances in the immigrant paradox pattern depending upon different developmental outcomes (5) and origins including ethnic and national origin differences (2), refugee (6), as well as documentation status (7). Over the past decade, research has suggested that when compared to other racial/ethnic groups, Latinx youth demonstrate high rates of both depression and anxiety (7). Documentation status appears to be a factor that may be contributing to varying patterns in mental health outcomes (8, 9).

Due to the vulnerabilities of undocumented individuals, however, few large-scale studies consider the impacts of legal status on mental health outcomes. An exception to this generalization is regards to a body of scholarship with undocumented college students who currently constitute an estimated quarter of a million students in the United States (10). While this growing and rich body of work has qualitatively explored the experience of both stress and resilience among these students (11, 12), there have been few studies to date, that have attempted to gauge potential rates of anxiety amongst them [(13), for an exception].

The aim of this study is to use a mixed-methods approach to not only identify the prevalence of anxiety in undocumented Latinx undergraduate students but to also provide insight into their lived experiences around coping with this issue. It also seeks to explore how several stressors (e.g., financial concerns, legal status) and potentially protective factors (e.g., peer support, faculty support) may contribute to anxiety levels.

### Conceptual Framework

We draw up an integrative model for the adaptation of immigrant-origin youth (14) that combines ecological (15) and risk and resilience frameworks (16). As immigrant families enter host societies, political, economic, and social factors within the contexts of reception (17) influence short-term adaptation as well as long-term developmental pathways for new immigrants and their children. These contexts of reception in which immigrant-origin children and youth settle present both resources and risks with significant implications for shaping developmental tasks, and psychological adjustment (14).

An ecological perspective suggests that interrelated contexts of development within which youth are embedded shape opportunities and have important implications for a number of developmental outcomes (14, 15). The decades-long context of a political stalemate around comprehensive immigration reform and legalizing DREAMers (18), an ever-intensifying deportation machinery (19, 20), and xenophobic public media messages (21) set the stage for the macrosystemic ecological context in which undocumented students face their college experience (14, 22). This macrosytemic context has shaped societal attitudes toward immigrants, resources, and the opportunity structure for students (14) and has implications for a host of outcomes including educational trajectories and psychological wellbeing (23). While recognizing the extraordinary resilience of undocumented youth, the stressors they are facing at this macrosystemic level place them at heightened psychological risk (12, 14, 23, 24).

At a more microsystemic level, optimally, educational settings can “bridge cultural distance” (25) serving as “sites of possibilities” (26) supporting credentialing, language acquisition, and life-long learning. Supportive relationships with peers and teachers serve a particularly important buffering function. These relationships are often integral to promoting academic motivation, supporting feelings of belongingness, and navigating the narrow educational pipeline for first-generation to college students living in unauthorized families (27).

### Literature Review

Anxiety disorders are one of the most common mental health disorders with ~29% of U.S. adults having one or more diagnosable anxiety disorders (28). The rates for immigrant Latinx adults who receive this diagnosis, on the other hand, are estimated to be roughly 15% (2). The lower rates of anxiety and mental health issues for immigrant Latinx give an indication that they may be faring better overall when compared to U.S. born Latinx; on the other hand, these rates may be in part a result of under-utilization of services and cultural and linguistic limitations of measures (21, 29). These studies, however, did not distinguish the differences in legal status among the groups under investigation.

A study of 281 first-generation Latinx youth ages 12–19, found that they face multiple challenges that include poor socioeconomic resources, the risk of behavioral problems, and low educational attainment (7) and that nearly 29% reported symptoms of anxiety. Others have found that Latinx students are more likely to suffer from mental health issues and lack access to mental health care than non-Latinx Whites and African Americans (30–32). Potochnick and Perreira (7) postulate that legal status had a significant effect on anxiety and other mental health issues for first-generation Latinx.

### Generalized Anxiety Disorder

Generalized Anxiety Disorder (GAD) is characterized by chronic excessive worrying about differing events and activities for at least 6 months (DSM-V). This worry is often difficult to control. Some have posited that this disorder is linked to uncontrollable and unpredictable aversive events although generally not traumatic or as severe as those events causing Post-Traumatic Stress Disorder (28). Additionally, people suffering from GAD are especially concerned about not being able to predict the future (28). Undocumented individuals may be prone to developing this anxiety disorder as they often feel unsure of the future due to their legal status (9, 33). As such, worrying about deportation and detainment of themselves or loved ones, financial issues, a general uncertainty may place these youths at risk of developing excessive worries that could lead to this anxiety disorder (34).

### Gender, Immigration, and Anxiety

It is well established that women typically report higher rates of anxiety than men (31 vs. 19%) of anxiety (35–39). Similarly, research suggests immigration impacts women differently than it does men and can be attributed to a number of contradictions between the home and host cultures (8). In particular, appropriate gender role behavior seems to be stronger for women who may be seen as “keepers of the culture” by attempting to uphold homeland traditions, language, and strong ethnic identity which can be used as a defense against the identity loss that may be experienced through the process of adjusting to a new culture (40, 41). This pressure for women to preserve the culture, as well as managing the conflicting messages received from the home country and family, and putting the needs of the family before their own can lead to maladjustment of immigrant Latinx women (39, 42).

Latinos, on the other hand, tend to report less anxiety than Latinas (35). Like Latinas, Latinos also have to contend with the traditional gender roles ascribed to them and often are seen as the protectors as well as the financial providers of the family (43). Males may cope in a differing way from women, acting in more self-reliant ways that fit with the social and cultural notion that males are expected to solve problems rather than seek help (44, 45). Role changes may occur as they adjust to the new culture, specifically if they leave home to attend college thereby finding themselves less able to provide for their family which can cause substantial stress as they try to negotiate their new roles (44).

### Undocumented College Students in Higher Education

Undocumented students face multiple challenges which often include being the first in their family to attend college, living in a home with mixed-status family members, as well as little financial help that leads many to report a feeling of stress, anxiety, and other psychological symptoms (22). Many of these undocumented students, however, often do not experience the full limitations of their status until the later years of adolescence as they are preparing to apply for college or begin to think about their future after high school (8, 46). Not only do undocumented students face the transition into adulthood, but they also face a transition into “illegality” (46). Undocumented students' legal status may have less impact during their childhood as they are guaranteed an education Plyer vs. Doe in K-12 institutions, but it becomes a defining feature in late adolescence and into adulthood as they realize the limitations of their status and are unable to fully participate in the college experience (46).

Indeed, various studies have captured the difficulties undocumented students experience as they navigate higher education [e.g., (8, 22, 47, 48)], with college affordability (9), discrimination (49, 50), and a lack of safe spaces (47). Similarly, other studies have illustrated the chronic fear of deportation (11), uncertainty about their futures and constraints of working, studying and commuting (8, 22), which appear to be contributing factors to negatively impact undocumented student's psychological wellbeing [e.g., (8, 13)]. However, few studies, have explored how these factors are linked to impacting their anxiety rates using a mixed-methods strategy.

Notably, scholars argue the importance of creating safe spaces, where undocumented students can find support and resources, to alleviate the risk associated with an undocumented status in college campuses [e.g., (22)]. Studies revealed that the fear of any negative reaction from peers or institutional agents (e.g., faculty, staff), such as exposing their legal status, tends to create constricted social networks for undocumented students (12, 22). Similarly, scholars have argued that a lack of support from institutional agents and dealing with microaggressions (e.g., insensitive comments about their legal status (49) may make these students apprehensive about seeking help from peers or professors (8, 22). This is important as research has noted that little social support and fewer close relationships are linked to lower mental health (51). Undocumented students, at the same time, have reported college success by drawing on their resiliency, which they attribute to learning from their institutional and community mentors (12, 52). Thus, if undocumented colleges students are unable to access a larger social network due to their legal status, they may be at a higher risk of experiencing anxiety.

Additionally, in most states, there are policies and institutional practices that may limit access and success to higher education for undocumented students with limited financial resources to help enroll and pay their tuition (10, 49). Nonetheless, eligible undocumented students are granted a 2-year work-permit and relief from deportation when Deferred Action for Childhood Arrivals (DACA) was passed on June 15, 2012, by the Obama administration. Scholars have noted that DACA helps alleviate some of the challenges of being undocumented, which help improve their educational journey (53). While many of these students have reported educational benefits (e.g., being eligible for jobs and internships) to DACA, scholars argue that these “short-term” benefits do not provide a pathway to citizenship, which Gonzales et al. (8) suggest would help address college affordability and vulnerability to enactments of xenophobic policies (8, 54). Indeed, the Trump administration has continually undermined DACA demonstrating the vulnerability of this short-term solution (55, 56).

In addition, young adulthood is an age of particular vulnerability for mental health challenges as nearly three-quarters of lifetime psychiatric disorders emerge in adolescence and early adulthood (57). College campuses across the country are seeing an increase in serious psychological issues such as depression, suicidal ideation, and alcohol abuse (31, 58). In an annual report by the Center for Collegiate Mental Health (59), over half of students indicated a current concern related to anxiety. Untreated mental illness is a growing concern for campuses across the country with significant implications for academic success, productivity, substance use, and social relationships (60).

Today, with the recent intensification of explicitly anti-immigrant federal policies (55) as well as post-election anti-immigration climate (56) these issues are of pressing concern (61). Emerging qualitative data has documented that “politically difficult times” have increased reported anxiety and fear from undocumented students [(62), p. 273]. Similarly, other studies have noted that political awareness about anti-immigrant sentiment during the 2016 elections caused Latinx youth to feel anxious, fearful, disgusted, and angry. To date, however, we know little about the undocumented students' potential rates of symptoms as these students are often invisible either because they are fearful or because they are overlooked (10).

### Aims

The aim of this research is to fill the gap in the literature by examining the presence of anxiety among Latinx undocumented undergraduates in a sample of California college campuses. One aim of this study was to uncover how undocumented Latinx undergraduates' legal status may influence their levels of anxiety. It also sought to examine how stressors (financial concerns, deportation worries) and potential protective factors (peer support, faculty support) influence their anxiety. Lastly, it sought to shed light on these students' experiences. An embedded mixed-methods research design (63) was conducted in which the primary strand of research was quantitative (consisting of forced-choice survey questions) with select open-ended qualitative questions embedded into the survey in order to provide some phenomenological insights in the undocumented student experiences. Our research questions consisted of:

**Quantitative**. 1- What are the self-reported levels of anxiety among undocumented Latinx undergraduates in California? Is there a gender difference in the moderate and severe ranges? 2- How do stressors (e.g., fear of deportation, financial concerns) and potentially protective factors (e.g., peer support, instructor support) impact the level of reported anxiety of undocumented Latinx undergraduates?

**Qualitative**. 1- How do undocumented Latinx undergraduates describe their stress in relation to being undocumented college students? 2- In what ways might these stresses and challenges contribute to anxiety?

We hypothesized that undocumented Latinx undergraduates would report high levels of anxiety in comparison to the norm group. We also anticipated that women would report higher levels of anxiety than men. Additionally, we expected that the unique stressors (financial concerns and fear of deportation,) would increase anxiety, while social supports (peer and instructor) would reduce reported levels of anxiety.

## Methods

### Data Collection Strategy

Due to their stigmatization and invisibility, undocumented Latinx undergraduates are an especially “hard-to-reach” population (64). Thus, novel strategies were employed to reach the survey sample. The *[BLINDED] Project* was initiated in response using innovative strategies to gain access to this population (10). Our research team, consisting of three diverse Principal Investigators and a team of diverse graduate students. We partnered with 10 community organizations working locally and nationally with undocumented students and the community as well as an Undocumented Student Advisory Board, and a Faculty Expert Advisory Board. We collaborated on the design and piloting of the survey with our advisory team. The primary recruitment method for the project was through a web portal and a strong, multi-platform social media campaign (e.g., Facebook, Twitter, Instagram) that specifically focused on providing information about undocumented issues as well as providing them the opportunity to have their voices heard by participating in the study. Two months before the project was launched, a website was designed, providing resources and information for undocumented students. We let potential participants know that we wanted to hear from them about their experience. The advisory members worked with our research team on an active social media campaign to widely send out information about the survey across the undocumented community and college resource networks. We also recruited through the use of flyers, attending Deferred Action for Childhood Arrival (DACA) college events, and word-of-mouth. Data was collected from January through July of 2014.

To qualify, participants had to meet the following eligibility criteria: 1- Be between 18 and 30 years of age, 2- Identify as an undocumented, DREAMer, or DACAmented college student, and 3- Have been enrolled as an undergraduate student in the college in the past year. In addition, due to the vulnerability of the population, participants were assured anonymity; consent for participation was a simple checkmark before starting the survey. Approximately 85% of the surveys were collected via Qualtrics; the remaining surveys were collected through paper and pencil surveys and entered into SPSS. Once the participant completed the survey, if they wished to receive a $20 gift card, they were prompted to send us an email to receive the card. A data control protocol was implemented in order to reduce the number of mischievous survey data (65) (e.g., 80% of survey complete; language in synch with country of origin) and each survey was checked by a group of research team members. Once the survey was deemed valid each participant received the gift card in return for her or his participation. The data was then transferred from Qualtrics to SPSS and all identifiable data including email and IP address was deleted from our server. This project was approved by the Internal Review Board (IRB#13-001614).

### Participants

The overall [BLINDED project name] sample consisted of 909 diverse participants from 34 states; approximately half of the sample came from California. California has the largest population of undocumented immigrants in the nation with a current estimate of between 74,000 undocumented immigrants enrolled in college (66). Further, it affords undocumented college students benefits, such as the Development, Relief, and Education for Alien Minors (DREAM) Act (which was a policy in California before the DACA^1^ program was implemented) which allows undocumented students who meet certain criteria to apply for and receive private scholarships, state-funded financial aid, university grants, community college waivers and Cal Grants that are not accessible in other states. As such, for this article, we restricted our sample to a sub-sample of 486 self-identified undocumented (including DACA, expired work or student visas or under review) Latinx undergraduates from California.

Females constituted 54% of the sample. Participant's ages ranged from 18 to 30 years of age with a mean of 21.60 (*SD* = 2.69). Participants came from a variety of Latin American countries, with the largest percentages coming from Mexico (84.1%), Guatemala (3.0%), Peru (2.6%), El Salvador (2.2%), and Colombia (1.8%). Age of entry into the U.S. ranged from 0 (birth to 11 months) to 16 years of age (*M* = 6.22, *SD* = 4.34). Forty five percent of participants attended a 2-year public community college or university, 48.8% attended a 4-year public college while 3.8% attended a private university. Roughly 62.7% of participants identified themselves as DACAmented students with temporary protected status of deportation. The majority (95%) of the sample had a total household income of $49,999 or less with the largest percentage (28.1%) having a total household income between $20,000 and $29,999 per year.

### Measures

#### Quantitative

##### Financial concern

A brief version of the financial concerns scale was used [see (67), for original categories] to capture undocumented college students' concerns, for example, with financing education, expenses relating to textbooks and supplies, as well as medical/dental expenses. The 6 items were rated on a 5-point Likert-style scale (1 = not at all concerned to 5 = extremely concerned). The mean for all items was computed and higher scores indicated greater financial concerns for each domain. Internal reliability for this sample is α = 0.83.

##### Concerns with deportation

Two separate items were used to assess the level of worry participants felt about deportation/detainment. The questions included were “How often are you worried that you might be detained or deported?” and “How often are you worried that your family members or friends might be detained or deported?” Responses were rated on a 4-point Likert-style scale (0 = never to 3 = most of the time). A mean or sum was not created as each item was used to assess the worries separately.

##### Peer support

Peer support concerning legal status was assessed using 4 items on a 5-point Likert-style scale (1 = strongly disagree to 5 = strongly agree). Sample items included, “I feel safe sharing my legal status with my friends” and “My friends support me around my legal status.” The mean of all items was calculated with higher scores indicating greater levels of positive relationships with peers. Internal reliability for this sample is α = 0.80.

##### Institutional agent support

Different patterns of positive relationships and support from instructors and staff on campus regarding participants' undocumented status were captured using the 9-item Instructor Relationships scale (22), rated on a 5-point Likert-style scale, (1 = strongly disagree to 5 = strongly agree). A sample item included, “There are [instructors/staff] I can approach if I have a personal problem.” The mean was calculated with higher scores indicating greater levels of positive relationships with instructors and staff on campus. Internal reliability for this sample is α = 0.92.

##### Generalized anxiety

The 7-item Generalized Anxiety Scale (GAD-7) (68) was used to assess self-reports of generalized anxiety disorder. In a norm sample (*N* = 2,982) consisting of 80% White, 8% African American, and 9% Latinx meeting diagnostic GAD criteria for 6 months, construct and criterion validity of this measure was established comparing self-report GAD to “independent mental health professionals' diagnoses, functional status measures, disability days, and health care use” (p. 1092) demonstrating good reliability and validity (68). Participants provided responses to the prompt, “Over the last 2 weeks, how often have you been bothered by the following problems?” with sample items such as “Trouble relaxing” or “Not being able to stop or control worrying.” Answer choices were rated on a 4-point Likert-style scale (0 = not at all to 3 = nearly every day). The raw scores on the 7 items are summed with a score ranging from 0 to 21 to assess the severity of symptoms with 0-4 = minimum levels of anxiety; 5-9 = mild levels of anxiety, 10-14 = moderate levels of anxiety, and 15-21 = severe levels of anxiety. A cut-off score of 10 or greater has been established to demonstrate the highest sensitivity (89%) and specificity (82%) (68). The use of the scale has been found to be valid and suitable for use with Latinx in the U.S. who speak both English and Spanish (69). Internal reliability for this sample is α = 0.91.

#### Qualitative

As part of the survey, 3 open-ended qualitative questions were included in the survey. One asked about how their college experience had changed since receiving DACA status; another asked about the challenges of undocumented status and the last asked for what advice they would offer colleges to better serve undocumented students. For this contribution, we analyzed responses to the questions “Being an undocumented student can be stressful. What are the biggest challenges you face as an undocumented student?” Ninety-six percent of participants provided a written response that ranged in length from a minimum of 5 words to multiple paragraphs.

### Researcher Positionality

The research team for this project was made up of a diverse group of first- or second-generation immigrants, many but not all of whom are Latinx; some of the members of the research team were DACAmented or undocumented or from mixed-status families (22). The senior author is a first-generation immigrant who has done extensive research, training, and advocacy work in the immigrant and undocumented community. The second author is also a first-generation immigrant who was previously undocumented herself and is part of a mixed-status family.

### Analysis

#### Quantitative

To examine our first research question frequencies were used on forced-choice survey items from the measures listed above. To examine whether there were differences between men and women in their reported levels of anxiety, we employed an independent samples *t*-test. To examine how stressors and potentially protective factors predict anxiety, we conducted a four-step hierarchical multiple regression, using a sequential approach. Correlations were first run on all variables of interest (see Table 1 for correlations). The first step of the regression included gender and age as control variables. In the second step, worries related to family or self-deportations or detainment was entered. In the third step, concerns about finances were entered into the model. In the final step, undocumented peer support and instructor support were entered.

**Table 1:** Means and correlations of variables (*N* = 486).

	***1***	***2***	***3***	***4***	***5***	***6***	***7***	***8***
1. Gender	__							
2. Age	−0.049							
3. Fam deportation	0.160^**^	−0.065						
4. Self-deportation	0.108^*^	−0.04	0.421^**^					
5. Financial concerns	0.106^*^	−0.065	0.156^**^	0.194^**^				
6. Undoc peer support	0.196^**^	−0.089	0.165^**^	0.028	0.152^**^			
7. Instructor support	0.149^**^	0.041	0.246^**^	0.042	0.127^**^	0.435^**^		
8. Anxiety	0.059	−0.144^**^	0.168^**^	0.150^**^	0.294^**^	−0.043	−0.066	
Mean	21.52	1.74	1.34	3.15	3.68	3.69	7.21	7.65
*SD*	2.69	1.04	0.96	0.98	0.91	0.81	5.42	4.35

*Gender variable coded as 0 = Male, 1 = Female*.

* *p < 0.05*,

** *p < 0.01*.

#### Qualitative

We employed both inductive and deductive approaches toward our qualitative analyses. We began, inductively by reading through all the responses to determine what codes emerged from the data (70). That initial inductive coding yielded ~20 codes.

To achieve interrater reliability, two researchers individually read and coded a random subsample of 98 (20%) responses. We coded using a binary coding scheme for each theme with 0- code not present and 1- for code present. We then calculated the interrater reliability using Cohen's kappa. After initial coding, the two coders met twice to discuss, revise, and come to a consensus before final coding. Final coding kappa ranged from 0.81 to 0.97 on coding themes and categories meeting criteria for almost perfect agreement (71).

After coding was completed, codes were later collapsed into themes that aligned with deductive categories consistent with our quantitative model (e.g., financial concerns, deportation concerns, and social supports, and anxiety). In addition, moving beyond our predictive model, we also present the most prevalent themes that emerged in order to further shed light on the lived experience of undocumented college students (see **Table 3** below for code definitions).

## Results

### Quantitative Findings

#### Reported Generalized Anxiety

Descriptive analyses revealed that 32% of the sample met the cutoff criteria (10 points or greater) and, as such, were self-reporting symptoms of generalized anxiety disorder at the moderate to severe level as indicated by the GAD-7.

#### Gendered Findings

Turning to gender patterns, we found that 28% of males and 35% of females met the cutoff criteria of 10. These numbers are striking when compared to the norming sample, in which 4% of males and 9% of females met the criteria for generalized anxiety disorder (68). Thus, the rates of GAD in this undocumented college sample is 7 times higher for males and nearly 4 times higher for females than that of a general population sample.

Independent samples *t*-tests comparing gender differences ratings of self-reported anxiety using the GAD-7 cutoff of 10 points or greater (indicating moderate to severe levels of anxiety) showed a significant difference between Latina (*M* = 14.61, *SD* = 3.70) and Latino (*M* = 12.41, *SD* = 2.91); *t*_(119)_ = −2.98, *p* < 0.05 undocumented students. Thus, as with other populations, undocumented Latinas appear to suffer higher levels of generalized anxiety than their male counterparts.

#### Stressors and Social Support Predicts Anxiety

The multivariate regression was then conducted to test the effects of stressors and social supports on symptoms of anxiety. Each step of the regression analyses resulted in an increase in *R*^2^. In the first step of the regression, age was a significant predictor for anxiety symptoms explaining 2% of the variance. The older the participant the lower their anxiety levels. In the second step, age and worries about family deportation significantly predicted anxiety explaining 5% of the variance. In the third step, age, concerns about self and family deportation with the additional financial concerns together significantly predicted levels of anxiety explaining 12% of the variance. In the final model, age, worries about family deportation or detainment, financial concerns, and instructor support together significantly predicted 14% of the variance in reported levels of anxiety *R*^2^ Δ = 0.02, F change (2, 469) = 5.32, *p* < 0.05. This suggests that while family and friend deportations predicted anxiety, worrying about finances was still the leading contribution to anxiety. On the other hand, having a supportive institutional agent contributed to reduced reported anxiety levels (see Table 2 for regression results).

**Table 2:** Hierarchical regression analyses of stressors and potentially protective factors on anxiety.

**Predictor variables**	**β**	**Beta**	**SE B**
Step 1			
Age	−0.284^**^	-0.141	0.091
Gender	0.571	0.052	0.494
F	5.67^**^		
R2	0.023		
Step 2			
Age	−0.264^**^	-0.131	0.09
Gender	0.264	0.024	0.494
Fam Deportation	1.734^*^	0.116	0.743
Self-Deportation	1.226^*^	0.093	0.65
F	6.70^***^		
R2	0.054^**^		
Step 3			
Age	−0.237^**^	-0.118	0.087
Gender	0.052	0.005	0.479
Fam Deportation	1.434^*^	0.096	0.72
Self-Deportation	0.709	0.054	0.635
Financial Concerns	0.469^***^	0.26	0.08
F	12.57^***^		
R2	0.12^***^		
Step 4	1		
Age	−0.236^**^	-0.117	0.088
Gender	0.315	0.029	0.484
Fam Deportation	2.055^**^	0.138	0.738
Self-Deportation	0.493	0.037	0.633
Financial Concerns	0.496^***^	0.275	0.08
Peer Support	−0.071	-0.07	0.051
Instructor Support	−0.087^*^	-0.104	0.042
F	10.67^***^		
R2	0.14^**^		

* *p < 0.05*,

** *p < 0.01*,

*** *p < 0.001*.

#### Qualitative Findings

The responses emerging from the open-ended questions served to capture the lived experience of the anxieties undocumented college students live on a daily basis. Participants highlight the challenges they faced as well as their own accounts of how their status directly impacts their everyday experiences contributing to uncertainty and anxiety (see Table 3 for percent of codes).

**Table 3:** Deductive and inductive codes, definitions and percentages (*N* = 486).

**Code**	**Definition**	***n* (%)**
Financial	Concerns about finances, including funding tuition and daily living expenses for themselves and family	277 (57)
Deportation/Detainment	Concerns/fear about self or loved ones being deported/detained	31 (6)
Peer Support/Lack of	Mention of the importance of friends and lack of friendships/social support from peers	14 (3)
Institutional Agent Support/Lack of	Mention of the importance of professors or staff on campus and lack of social support from them	11 (2)
Anxiety	Feelings of anxiety and worry	69 (14)
Social Belonging/Exclusion	Mentions the importance of belongingness and their concern the role UndocuStatus has in social exclusion or marginalization	43 (9)
Hopelessness	Refers to feeling hopeless/discouraged	8 (2)
Uncertainty	Concerns about future; lack of control; inability to plan	13 (3)

*Percentages are based on how many times a code was present. Participants may have mentioned multiple things within their answer and each section was coded separately and according to the appropriate code*.

#### The Experience of Anxiety as an Undocumented Student

Participants in this study revealed the numerous ways their undocumented status negatively impacted their psychological wellbeing. Various responses captured how these students were grappling with “staying mentally, physically, emotionally healthy.” [Female, 26, 2-year public community college] More specifically, many of these students reported feeling “stress, depression, anxiety, feeling[s] of alienation.” [Female, 22, 4-year public institution] Many reported disruptions to their sleeping patterns, enjoyment in life, and thoughts about their future. As one student explained: “My stress as [a] student is that I can't sleep for the worries. I have my confidence goes down.” [Female, 23, 4-year public].

Many responses were specifically centered around student's difficulties focusing in school due to the anxieties related to their legal status. For example, a participant reported: “Waking up every day with the worry I have, and going to school and not being able to focus in class and this has affected my grades so badly.” [Male, 22, 2-year public community college] Others, similarly, described the stress of not having control over their lives, as noted by a 20-year old male attending a 2-year public community college: “When stress breaks in, I cannot do math anymore, even though I am a math lover…because of [the] possibility of being detained.”

The undocumented students in our study compared themselves to their documented peers: “We can't enjoy life because we might be in trouble and get deported. It's hard for a 19-year-old to not enjoy life while I see all my classmates having fun and they can concentrate afterward on schoolwork.” [Male, 19, 2-year public] Many articulated the multitude of stressors they juggled related to their legal status, including financial burden, deportation fears, and their sense of social exclusion.

#### Financial Concerns

The participant's qualitative responses were consistent with quantitative analyses—concerns about money was the most pressing challenge they reported (56% of respondents spontaneously brought issues related to financial pressures). A majority of responses were centered around participant's concerns for the lack of financial aid they are eligible for and the limits of their status in acquiring funding for their college tuition. One student explained: “Financial struggles are the greatest cause of my stress. I am constantly worried that I won't be able to pay to continue my education. I'm afraid of being stuck just because I can't get the financial aid I need.” [Female, 19, 2-year public community college] Similarly, another revealed the difficulties of finding financial assistance: “Some of the biggest changes I face as an undocumented would be applying for scholarships because most scholarships require citizenship.” [Female, 19, 4-year public] Notably, all of these participants lived in California with inclusive policies such as the California Dream Act; nonetheless, many came from high poverty homes with pressing financial concerns. As one student stated:

My state has the CA DREAM Act available to me. It has helped take away many financial burdens off my family's and my back. Before that, it was a big struggle to go to school because my parents were shouldering most of the cost for my school in addition to paying rent, utilities, and so on. [Female, 20, 4-year public].

These responses demonstrate that these students were not only worried about financing their education, and of not being a burden on their parent's finances; they also worried about contributing to their family's expenses and income. A 24-year old student attending a 2-year public community college described this concern: “I provide for my younger siblings' school supplies and clothes. I'm no longer their sister, I've become a second mom and when money is not enough, I have to place my school costs, including textbooks, as a second priority.”

As a result, it was not surprising that nearly three-quarters of participants worked full-time or multiple jobs in low-wage menial positions that exacted a toll on their school productivity, engagement, and psychological wellbeing (10).

[I worked] full time and attend school part-time since part-time tuition as all I was able to afford…. money has always been an issue. I attended community college first and then transferred out to a 4-year university, throughout this time I always had to depend on public transportation since I did not own a car. My body was always tired and felt heavy, I was mentally drained and suffered from depression. [Female, 27, 4-year public].

Despite attaining college status, just like their parents, undocumented students reported wage exploitation: “One of the challenges that…[I] face is the ability to pay for school. Finding a job is even more difficult. Having to raise a family on minimum wages almost seems impossible and sometimes doesn't seem worth it].” [Male, 20, 2-year public community college].

Notwithstanding multiple obstacles, these highly resilient students described how these impediments also served as motivation to help them succeed in spite of the odds. The participants' responses provide insights into their determination:

Financially one has to work two jobs due to our family needs … That constant concern of helping your family and at the same time going to school is an obstacle, but the appreciation for our obstacles moves us forward to succeed. [Female, 23, 4-year public].

#### Deportation Worries

Another worry that participants vividly articulated was their concerns around deportation or detainment. Notably, in the California sample, concerns around detention was reported less frequently than in the nationwide sample, a probable reflection of these states' UndocuFriendly policies (10). For some, this fear was present on a sustained basis. As a student explained: “I fear being deported, I am living in fear every day I go to school and go to work.” [Male, 18, 2-year public] Another wrote about their worries of not knowing who to trust: “Confiding with other people about my legal status. I don't because I fear if they ever get mad at me they could report me to border patrol.” [Male, 28, 4-year public] Not surprisingly, these fears place undocumented students in a heightened state of alert: “I fear every day being pulled over by the police only to show no license and have to deal with the humiliation and legal ramification.” [Male, 22, 4-year public].

We should note, that this data was collected prior to the 2016 election. At that time, interestingly, for students who were able to secure DACA, reports of worries about deportation of themselves plummeted; concurrently, however, the worries about deportation of close family members and loved ones went up (10). In a concise but telling response to the open-ended question about the greatest challenge of being an undocumented student, a respondent said: “[the biggest worry is] my family being separated because of our legal status.” [Female, 23, 4-year public] Another, worried about, “receiving the phone call that nobody wants to receive, informing you that a family has been deported.” [Male, 18, 4-year public] Some responses reflected practical concerns like: “I fear of my mother being deported, forcing me to leave school and take care of my siblings.” [Male, 21, 4-year public] These fears are not simply imagined as noted in this quote: “I constantly worry. [and now] both my parents have an order of deportation.” [Male, 20, 2-year public community college].

These quotes revealed the poignant, aching daily uncertainty that would have clear implications for generalized anxiety.

#### Potential Social Support/UndocuAllies

Undocumented Latinx, like many others, turn to social support as a form of coping (52). They often sought peers with a shared identity as a way to build a network to help them overcome the many emotional, academic, and social challenges they faced (72, 73).

##### Peer supports (and their limits)

The responses shed light on the role of peer supports that the quantitative findings were less sensitive in revealing. While some participants noted that their friends are the “only people that [they] can turn to” [Male, 20, 2-year public] others disclosed that they had difficulty finding support from undocumented peers, who “they can relate to.” [Male, 26, 2-year public community college] One student explained why this may be the case: “We are too afraid to “come out of the shadows” I believe that if we all gathered the courage to come out and stick together we could get more help.” [Female, 25, 2-year public community college].

Other students deliberately avoided developing a relationship with peers as they felt frightened and vulnerable by exposing themselves due to their legal status. As one student explained, [it is a challenge] “making friends because I fear they won't accept me for being here illegally in this country.” [Female, 21, 4-year public].

Even when undocumented students had friends to confide in, some noted that their peers struggled to understand the true limits of being undocumented. As one student eloquently explained:

A challenge is not being able to talk to someone, a peer to be more specific, that understands my situation. Most of my friends are there for me, but since none of my close friends are undocumented, if anything in the subject comes up, they are not sure how to react and often my feelings go unacknowledged. [Female, 20, 2-year public].

##### Instructor supports (and their limits)

The quantitative findings suggest, that having an adult (instructor or staff) member in the college community helped served to attenuate undocumented student self-reported anxiety. The qualitative responses, however, reveal that despite the great need for supportive institutional agents (12, 72) undocumented students were wary of seeking such help and disclosing their status because professors did not always understand their needs. Indeed, student responses to the open-ended prompts revealed few examples of instructor support.

Instead, several examples of vulnerability in the classroom and on campus emerged in the responses, as noted by a student: “I don't know who I can look up too.” [Male, 19, 2-year public community college] Many expressed concerns around to whom they could disclose or seek support. As a participant explained: “It is sometimes difficult to share your immigration status and people don't understand you, especially if those people are professors.” [Female, 23, 4-year public].

Another shared a similar sentiment of having difficulties connecting with instructors:

While my college tries to be supportive, there exist institutional barriers that prevent me from doing well academically. I'm a math major which historically, is not an area of study that Latinos, People of color go into. It is difficult to find support and professors that understand what it's like to be Undocumented. [Female, 18, 4-year private].

Several students reported that it was disheartening when professors assume that all students in the classroom are documented during discussions, inadvertently making their undocumented students uncomfortable when talking about immigration issues, as revealed here:

I face challenges from professors sometimes. In my sociology course, my professor singled me out because of my Mexican decent and asked for my take on deporting illegal immigrants to Mexico vs. keeping them here and watching our economy 'crumble.' It was extremely stressful. [Female, 20, 2-year public].

Others talked about the shame they felt around their undocumented status, blocking them from seeking out help: “It feels shameful asking for help because I don't feel ‘worthy' of receiving it.” This sense of stigmatization served as an impediment in their ability to open up and foster much needed supportive relationships. As this student went on to explain:

The biggest challenge I face as an undocumented student is dealing with the stigma and preconceived notions people have of the undocumented community. I feel as if there are very few people I can turn to and confide in regarding my status. [Male, 23, 2-year public].

As such, many of our participants seemed to have difficulty finding guidance from faculty mentors to maneuver the anxieties of being undocumented.

#### Seeking (But Failing to Find) a Place to Belong

Many of the participants spoke about their disconnection from their peers, college campuses, and American society more broadly. Many responses reflected that these undocumented college students felt “rejected,” “isolated” and simply did “not fit in.”

For some of our participants, this seemed to be linked to their inability to relate to their documented peers. For example, a female 18-year-old attending a 4-year public institution stated that her “biggest issue is being accepted by other documented peers.” Notably, undocumented students recognized that their inability to participate in “normal” societal milestones, such as the typical college experience, impacted their sense of belonging to American society, which has been noted by previous scholars (8, 74). For example, “being excluded from like studying abroad” [Male, 21, 4-year public], or not “finding internships to expand my abilities in my [field]” [Male, 22, 2-year community college] made them feel, unlike an “average student.” A male 22-year old attending a 2-year public community college student poignantly spoke to this point:

“The biggest stress factor is that I am basically American, I lived all my life here, I go to school here, work here but I'm not a citizen….[and I am] challenged in everything I do as a college student.”

Other students noted that Americans attitudes toward immigrants negatively impacted their sense of belonging in the U.S. As a 20-year-old male attending a 4-year private institution, reported, he perceived that “having people look at me as if I don't belong here [in the U.S]” was a primary influence to his stress.

Above all, the reminder that they can neither legally be here in the U.S nor become active members of society served to foster anxiety, frustration, uncertainty, and a sense of hopelessness among these participants. Some for example, feared their goals would never be achieved. Several spoke to concerns about whether their sacrifices were worth their efforts. As a female 25-year old attending a 4-year public institution explained, “[I] fear that earning a degree is a waste of time without obtaining legal status in this country.” Another student described this similar sentiment saying he “Sometimes fe[lt] like giving up because I think to myself, why get an education if I may not be able to work in this country legally?” [Male, 25, 4-year public].

Other responses captured their frustration about the uncertainty about their future.

Knowing that no matter how good I do on school, I might not be able to go to graduate school or have a job. I do not even qualify for DACA. Thus, after graduating I do not know what I am going to do. [Male, 19, 4-year public].

These statements (and many more like them) made by our participants, serve to show how these students are experiencing “worrying on a daily basis” and how this constant state of alert may be contributing to their elevated levels of anxiety.

## Discussion

An estimated quarter of a million undocumented college students attend our institutions of higher learning (22). These students face a highly stressful macrosystemic social context in which they face threats of deportation of themselves and their loved ones (11, 75) limited resources (54) liminality (76), and social disparagement and exclusion (74, 77). Previous rich qualitative studies have detailed both these students many challenges (11, 46, 52) as well as their resiliencies (73, 78) but, to date, given their relative invisibility across campuses and the hard to reach nature of this population, there is a limited survey data or sense of what might be the level or nature of some of their mental health challenges. Given the great stresses and uncertainties of life with which they must contend, it would be expected that they would report higher than average rates of anxiety. This study sought to provide insight into self-reported rates of anxiety in this population in comparison to a standardized norm population. We also hoped to provide a lens into the ways in which undocumented students described their challenges both to triangulate what we expected but also to reveal what we might have missed.

Not surprisingly, given the stresses, juggling acts, uncertainties, and liminality (33) these students described, nearly one-third of study participants reported moderate to severe levels of anxiety using a standardized measure to assess self-reports of generalized anxiety disorder. We found that 28% of Latinos and 35% of Latinas self-reported anxiety in the moderate to severe range above the cut-off score of 10; this rate is somewhat higher than findings conducted with a first-generation non-college Latinx population (7). The rates of self-reported anxiety for undocumented Latina students were 4 times that of the norm population; that of undocumented Latino students was 7 times higher as measured by the GAD-7 (68) in the moderate and severe ranges. We used a standardized and well-respected scale that has been established to be valid for use with Latinx in the U.S. (69) with a robust norming sample and a cut-off score of 10 with high sensitivity and specificity and as such can be quite confident that the self-reported anxiety levels are alarmingly high. While not a randomized sample (impossible to collect given the nature of this population) a contribution of this study is that we were able to collect a large sample of this hard to reach population across multiple campuses, providing some perspective on the issue. Further, the open-ended responses gave insight, succinctly stated in the quote of our title, describing what it is like to be an undocumented Latinx student: “waking up every day with the worry.” In particular, ongoing anxiety fuels served to contribute to anxiety (28).

The gendered finding was both predictable and surprising. Consistent with an array of other populations, when comparing males to females, in the range above the recommended cut off score of 10 (moderate to high self-reported anxiety), Latinas in this sample reported higher levels than Latinos (7, 38, 39, 79, 80). On the other hand, in comparison to the norm sample, Latinx reported a stunning anxiety rate 7 times higher than the norm group. Undocumented males and females, however, reported general similar rates of anxiety to one another. This is likely linked to the unique characteristics of the undocumented population. Specifically, undocumented Latinos are more likely to be the targets of detainment and deportation (81), which is likely to contribute to their heightened anxiety. As such, undocumented males appear to be at significantly higher risk than males in the general population for anxiety.

The quantitative findings revealed that several factors contribute to self-reported anxiety. Interestingly, age played a significant role; the older the student, the lower the reports of experiencing anxiety symptoms. Perhaps the transition to adulthood for these students (82) along with the transition from the relatively protected status, offered by Plyer vs. Doe in K-12 schools exposed and created unsupported spaces of higher education (46), is particularly stressful in the initial years. Responses to the open-ended question shed light on the compromised aspirations that some of these young people struggled with as to make sense of their futures. These responses also provided a sense of the frustrations of being cut-off from the “typical” on-campus college student experience, which often compromised their belongingness (8, 74).

The quantitative findings revealed that financial concerns were the single greatest contribution to levels of anxiety. This finding was echoed in the qualitative findings where the majority of participants reported that their ability to pay for college with limited (if any) financial aid benefits. Even in the California context, with the California Dream Act which provides for some tuition relief for in-state tuition, many juggled long hours of work while trying to concurrently focus on their studies, adding to their stress (8, 10, 22). This left undocumented Latinx students vulnerable and concerned with regards to being able to pay for tuition as well as daily living expenses for themselves and often for their families as well. The qualitative analyses, additionally, revealed the ways in which these financial worries translated into a life of constant juggling of roles of work and studying. Students expressed not only a constant fear of not meeting the gapping financial needs (to pay for school and life) but also of dropping balls in meeting their academic responsibilities.

Further, the quantitative findings showed that concerns with deportation contributed to negative symptomatology. Interestingly, concerns with the deportation or detainment of family and friends explained even more of the variance than did concerns about their own detainment or deportation though that too contributed. Previous analyses have found that counterintuitively students with DACAmented status report higher rates of anxiety than their fully undocumented peers (22). While they may feel more secure about their own protections from deportation, they worry about having exposed their undocumented family members by providing extensive residential and other personal information to immigration authorities in order to secure their own DACAmentation. They also reported bearing extra financial responsibilities as they sometimes were the only family member able to legally secure work (10). The qualitative findings shed light on the poignancy and the lived reality of these concerns. For some, there were practical worries of potentially assuming responsibility for siblings if a parent were deported. For others, the gnawing anxiety was around never knowing what might happen at any given moment.

In the quantitative analysis, we considered the potential for social supports from both peers and instructors to attenuate anxiety. We found that peer support failed to play a role in predicting the anxiety outcome; only a reported positive relationship with instructors had a positive effect on the anxiety outcome. The qualitative analyses provided some insights to help interpret these findings. While a few participants spoke of the importance of peers, anxiety around revealing status, shame, and feeling misunderstood, all served to interfere with reaching out to peers and making friends. As such, the limited social peer network upon which to drew their support, seemed to contribute to a sense of disconnection.

Notably, qualitative responses demonstrated that many undocumented students reported feeling like they did not belong (and in some cases were actively excluded) both from their college campus as well as to American society. Specifically, students reported that their inability to participate as an “average” college student and have access to a network of peers made them feel not included. This can be particularly concerning as these student's campus belonging has been established to be vital for an array of outcomes including academic self-efficacy, intrinsic motivation, as well as sense of social acceptance (83).

Participants also frequently expressed around feeling excluded from American society. And yet, “the need to belong is a powerful, fundamental, and extremely pervasive” social motivation (84) and human need (85). Humans are a social species who long to belong across a variety of domains including kinship groups, schools, places of work, as well as of course, in the nation-state (86). For members of stigmatized groups, however, a sense of social belonging is routinely compromised and at-risk (87) and is linked to its converse—social exclusion (86, 88, 89). We would argue, multiple forms of social exclusion, restrict not only access to resources but also can have negative implications for psychological wellbeing (8, 74).

The quantitative analyses revealed that social support provided by instructors served a buffering role for self-reported anxiety. The qualitative findings showed, however, that instructors were not always as sensitive as they could be in their interactions with their undocumented students or they simply did not understand their needs. Further, these students sometimes did not reach out as they felt shame and were uncertain who to trust. Previous analyses from this study, focusing on the open-ended question asking for advice to colleges to better serve these students, provided insights into tangible ways institutions could better support their students including providing safe spaces for them, training faculty and staff about undocumented student needs, providing up-to-date financial aid information, as well as providing culturally responsive counseling for them. Thus, this study builds on previous work adding insight into both the importance and gaps in social support for this population (73).

## Study Limitations

The nature of this study renders population-based random sampling impossible. As such, as with all non-randomized samples, findings cannot be generalized. Students from 4-year colleges/universities are over-represented relative to community college students which are of concern as undocumented students are more likely to begin college in 2-year settings (54). The sample for this article was restricted to California, which is a particularly “UndocuFriendly” state affording more rights and benefits to the undocumented population relative to other states. We might expect that the stressors and anxiety levels may be even more elevated in other states (particularly “UndocuHostile” like Arizona, Georgia, or South Carolina). Further, this study was conducted prior to the current presidential administration which has placed additional burdens on this population by making anti-immigration sentiments a center of its platform (90, 91). Since then both deportations (92, 93) and “unabashed xenophobia” (94) have arisen, which are likely to be adding to anxieties for these students. As such, these findings are probably an under-count of current anxiety rates across the country for this population.

Other limitations should also be considered. Undocumented Latinx college students who were less engaged, distrusting and anxious are less likely to participate. It is likely that the students who participated in this study are amongst the most active and open about their status, and most connected to the undocumented community. Additionally, the survey was all self-reported and is subject to response bias, and did not include gender-inclusive terminology (i.e., “Latinx^2^” (95, 96). Lastly, resources allowing, more qualitative questions would have been included in the survey allowing for a richer interpretation.

Future studies should include more of a balance of quantitative and qualitative questions. Further, future research in this area should work to better understand the range of mental health problems (including depression, for example) faced by this population. Future studies should expand beyond Latinx with more diverse immigrant backgrounds and include age equivalent comparison samples.

## Conclusion

This study has provided perspective not only on the potential prevalence of anxiety amongst undocumented Latinx college students but also what may be contributing to fuel these elevated states of anxiety. As these students now constitute an estimated 2% of our population, understanding their needs as they make this difficult transition is an important first step in serving them (22).

An important, practical implication for institutions of higher education is to be more aware of the variety of challenges faced by their undocumented students. One of the biggest challenges faced by college campuses is their increase in psychological services. Just as their campuses are increasingly diverse with 20% being foreign-born or first-generation, the mental health needs of their student body are increasingly broad and multicultural (58). It is imperative that college counseling centers and college administration pay attention to this population and work together to develop programs to assist undocumented students in ways that may be more culturally sensitive and relevant.

## Data Availability Statement

The de-identified data supporting the conclusions of this article will be made available by the authors, without undue reservation.

## Ethics Statement

The studies involving human participants were reviewed and approved by The Institutional Review Board, of University of California, Los Angeles. The patients/participants provided their written informed consent to participate in this study. Written informed consent was obtained from the individual(s) for the publication of any potentially identifiable data included in this article.

## Author Contributions

CS-O was the PI for the study and was principally responsible for developing and piloting the survey and directing the research team for the research project. She was primarily responsible for writing the Introduction, Literature Review, Methods, and Discussion sections. GL was principally responsible for the writing of the Results section. All authors contributed to the article and approved the submitted version.

## Conflict of Interest

The authors declare that the research was conducted in the absence of any commercial or financial relationships that could be construed as a potential conflict of interest.

## Footnotes

^1^Deferred Action for Childhood Arrivals 2012. To be eligible for DACA, individuals are required to: (1) be between the ages of 15 and 30 as of June 15, 2012, (2) have come to the U.S. before the age of 16, (3) have spent at least five continuous years in the U.S., (4) be attending high school or have a high school diploma (or equivalent) or a veteran of the U.S. armed forces or Coast Guard, and (5) have not been convicted of a felony or significant misdemeanor, and do not pose a threat to public safety or national security. DACA grants temporary relief from deportation, allows for a drivers' license to be issued and provides work authorization for a period of 2 years, subject to renewal.

^2^The term *Latinx* has rapidly become a trend on the Internet, social media platforms and among research scholars. The term was developed to become a “gender-neutral label for Latino/a and Latin@... to disrupt traditional notions of inclusivity and shape institutional understanding of intersectionality” (95, p. 302).
